# Dapagliflozin: Cardiovascular Safety and Benefits in Type 2 Diabetes Mellitus

**DOI:** 10.7759/cureus.1751

**Published:** 2017-10-05

**Authors:** Fatima Saleem

**Affiliations:** 1 Internal medicine, King Edward Medical University Lahore, Pakistan

**Keywords:** sodium-glucose co-transporter 2 inhibitors, dapagliflozin, diabetes mellitus, cardiovascular, sglt2 inhibitors

## Abstract

Sodium-glucose co-transporter 2 inhibitors (SGLT2is) such as dapagliflozin, canagliflozin, and empagliflozin, are a promising new therapy in the treatment of type 2 diabetes mellitus (T2DM). SGLT2is can effectively reduce hyperglycemia thus improving glycemic control and they offer some beneficial effects on the cardiovascular (CV) system which can benefit patients with heart failure in addition toT2DM. The United States Food and Drug Administration requires new diabetes mellitus therapies to show a CV safety profile. Empagliflozin was the first SGLT2i that, when added to the standard of care for patients withT2DM at high risk for CV events, showed improved CV outcomes including reduced deaths from CV causes. Evidence also exists in favor of dapagliflozin for use in patients with T2DM with CV risk factors and heart failure. This review focuses on the effects, safety, and benefits of dapagliflozin on the CV system. Clinical trials have shown that dapagliflozin improves glycemic control without variation. It is safe and well-tolerated in the general population including older patients and those with high-risk CV factors or preexisting CV disease. There may be a renal protective role by an unknown mechanism. Dapagliflozin also lowers blood pressure due to its natriuresis effect. It improves levels of visceral fat and reduces body weight, and thus ameliorates metabolic syndrome. Dapagliflozin reduces oxidative stress and may delay atherosclerosis. Recent findings indicate SGLT2is may also reduce the atrial natriuretic peptide levels. Additional trials are required to validate these benefits and further evaluate if these are class effects. Trials such as DECLARE-TIMI58 are ongoing to evaluate the CV outcomes of dapagliflozin. More research is needed to design better antihyperglycemic regimes with clinical benefits in addition to good glycemic control.

## Introduction and background

Sodium-glucose co-transporter 2 inhibitors (SGLT2is) are a new class of oral antihyperglycemic medication (OAM) shown to be safe and beneficial in cardiovascular disease (CVD) including heart failure [1,2]. SGLT2is have gained popularity given the available antihyperglycemic agents are less efficacious and may be harmful in patients with type 2 diabetes mellitus (T2DM) suffering from heart failure [2]. Initial treatment in patients with T2DM is lifestyle management including weight reduction, diet modification and increased physical activity. Even modest weight loss can improve glycemic control. If drug treatment is required, metformin is the initial therapy [3]. However, some patients are intolerant to metformin due to the side effects such as diarrhea (in about 10%) or renal impairment. SGLT2is are recommended in these patients [3]. Dapagliflozin, canagliflozin, and empagliflozin (i.e., three available SGLT2is) are effective in improving glycemic control [3,4]. The expected reduction in glycosylated hemoglobin (HbA1c) varies and is approximately 0.5% to 1.0% [5] for SGLT2is. Additional benefits of SGLT2is include a reduction in body weight and systolic blood pressure [2,3,6,7]. Other cardiovascular (CV) effects include reduced arterial stiffness [2]. Common adverse effects of SGLT2is are urinary and genital tract infections in a small proportion of users [3,8]. Nevertheless, more data are warranted to explore the advantages, side effects, and mechanism of action of this class of antihyperglycemic agents.

SGLT2is affect hyperglycemia and lower blood pressure (BP) by competitively blocking the SGLT2 receptors in the proximal convoluted tubules in the kidneys [5], preventing the reabsorption of filtered glucose and sodium, resulting in glycosuria and natriuresis [2]. SGLT2is reduce preload and afterload, cause volume contraction, and reduce BP (1 to 2 mmHg) due to osmotic diuresis and natriuresis physiological effects [2,7]. This provides cardiorenal protection. Besides a reduction in albuminuria (30% to 40%), an acute dose-dependent reduction in the estimated glomerular filtration rate by approximately 5 mL per minute is also noticed [7]. It has also shown a blood pressure lowering effect in normotensive people without diabetes and young patients with uncomplicated type 1 diabetes mellitus, perhaps due to changes in plasma volume as well as reduced arterial stiffness [7]. The natriuretic effect (and resultant osmotic diuresis) of SGLT2is could potentially be beneficial in patients with CVD, especially those with heart failure, thereby, distinguishing SGLT2is from the other OAMs [2,7].

Several clinical trials suggest the beneficial role of SGLT2is in patients with T2DM. Tang, et al. suggest the three new SGLT2is are not linked with an increased risk of all-cause mortality and CV outcomes when used to treat patients with T2DM. Randomized controlled trials (RCTs) of appropriate size are required to generate more information [9]. Many large RCTs are in progress to understand the CV outcome of SGLT2i therapy. As diabetes progresses, triple therapy is often required including metformin and two other agents (e.g., pioglitazone, sulphonylurea, glucagon-like peptide-1 inhibitor analogs, meglitinides, and glucose absorption inhibitors) [5]. A meta-analysis shows that adding SGLT2i to metformin in T2DM management is more efficacious compared to adding other antihyperglycemic agents; SGLT2i with metformin significantly improved HbA1c, body weight, and BP [8]. This meta-analysis is based on patients with T2DM who have inadequate disease control via lifestyle interventions and metformin. Of these, half of the patients were treated with SGLT2i and nearly half were treated with a non-SGLT2i combination (e.g., glimepiride/linagliptin/sitagliptin/glipizide) as an add-on to metformin [8]. Major findings of this meta-analysis are presented in Table 1.

**Table 1:** Major findings of the meta-analysis of six RCTs after 24 and 52 weeks with findings of the 104-week trials (total three trials of 104 week = two from the previous 6 RCTs + 1 new). _Dose Regimens: EMPA (25 mg/day), CANA (300 mg/day), and DAPA (up-titrated 2.5 to 10 mg/day). _ _CANA: canagliflozin; CI: confidence interval; DAPA: dapagliflozin; DBP: diastolic blood pressure; eGFR: estimated glomerular filtration rate; EMPA: empagliflozin; FPG: fasting plasma glucose; HbA1c: glycosylated hemoglobin-A1c; HDL: high-density lipoprotein; LDL: low-density lipoprotein; RCT: randomized controlled trial; SBP: systolic blood pressure [8]._

Parameter/duration	No. of RCTs	No. of participants	Mean difference [95% CI]	Significance level	Heterogeneity
HbA1c (%)					
After 24 weeks	6	4489	-0.00 [-0.02, 0.11]	P = .22	90%
After 52 weeks	6	4507	-0.11 [-0.18, -0.04]	P < .00001	54%
After 104 weeks	3	2707	-0.16 [-0.21, -0.08]	P < .00001	22%
FPG					
After 24 weeks	2	1142	-0.65 [-1.34, 0.04]	P = .06	80%
After 52 weeks	5	4188	-0.65 [-0.94, -0.35]	P < .0001	84%
After 104 weeks	3	2707	-0.72 [-0.86, -0.58]	P < .00001	0%
Body Weight					
After 24 weeks	5	3274	-3.98 [-4.68, -3.28]	P < .00001	82%
After 52 weeks	6	4147	-3.87 [-4.94, -2.80]	P < .00001	95%
After 104 weeks	3	2707	-3.53 [-4.86, -2.21]	P < .00001	92%
SBP					
After 24 weeks	1	1545	-5.60 [-6.91, -4.29]	P < .0001	-
After 52 weeks	5	4276	-4.88 [-5.66, -4.10]	P < .00001	23%
After 104 weeks	3	2707	-5.33 [-6.29, -4.36]	P < .00001	0%
DBP					
After 24 weeks	1	1545	-2.40 [-3.50, -1.30]	P < .00001	0%
After 52 weeks	4	4008	-2.38 [-2.93, -1.84]	P < .00001	62%
After 104 weeks	3	2707	-2.55 [-3.19, -1.91]	P < .00001	0%
eGFR					
After 24 weeks	2	1064	2.32 [-0.14, 4.78]	P = .06	34%
After 52 weeks	4	2139	3.43 [1.65, 5.21]	P = .00002	77%
After 104 weeks	2	1051	0.26 [-6.12, 6.63]	P = .94	80%
LDL % change					
After 24 weeks	1	970	9.00 [3.47, 14.53]	P = .001	-
After 52 weeks	3	2449	2.47 [0.25,4.68]	P = .03	99%
After 104 weeks	1	967	8.00 [2.07, 13.93]	P = .008	-
HDL % change					
After 24 weeks	1	967	7.50 [4.87, 10.13]	P < .00001	-
After 52 weeks	3	2449	6.89 [5.82, 7.96]	P < .00001	97%
After 104 weeks	1	1067	9.30 [6.65, 11.95]	P < .00001	-

SGLT2i as an add-on therapy to metformin treatment was found to be associated with significantly improved outcomes compared with non-SGLT2i combinations for up to two years of treatment. Improvement in the change from baseline in HbA1c was significantly more in SGLT2i therapy than in non-SGLT2i combinations in the long-term treatment [8].

In the distinct EMPA-REG OUTCOME study (which enrolled and treated >7000 patients; see Table 2), patients with T2DM with high risk for CV events when treated with empagliflozin had a significantly lower rate of death from any cause [9-11].

**Table 2:** Efficacy outcomes from EMPA-REG OUTCOME trial*. _*From Oral, 2016 [1]; Zinman, et al., 2015 [10]; and Fitchett, et al., 2016 [12]. ^a)^Pooled analysis of empagliflozin 10 and 25 mg. ^b)^Death from CV causes, non-fatal MI, or non-fatal stroke: primary outcome. ^c)^For noninferiority. ^d)^For superiority. ^e)^Death from CV causes, non-fatal MI, or non-fatal stroke, or hospitalization for HF: secondary outcome. ^f)^Excluding silent MI. _ _CV: cardiovascular; HF: heart failure; MACE: major adverse cardiovascular events; MI: myocardial infarction._

CV outcome	Placebo (N = 2333) n (%)	Empagliflozin (N = 4687) n (%)	Hazard ratio (95% CI)	p-value
Three-point MACE	282 (12.1)	490 (10.5)	0.86 (0.74-0.99)	<0.001 0.04
Four-point MACE	333 (14.3)	599 (12.8)	0.89 (0.78-1.01)	<0.001 0.08
All-cause mortality	194 (8.3)	269 (5.7)	0.68 (0.57-0.82)	<0.001
CV death	137 (5.9)	172 (3.7)	0.62 (0.49-0.77)	<0.001
Non-fatal MI	121 (5.2)	213 (4.5)	0.87 (0.7-1.09)	0.22
Non-fatal stroke	60 (2.6)	150 (3.2)	1.24 (0.92-1.67)	0.16
Hospitalization for HF	95 (4.1)	126 (2.7)	0.65 (0.50-0.85)	0.002
Hospitalization for HF or CV death	198 (8.5)	265 (5.7)	0.66 (0.55-0.79)	<0.001
Hospitalization for or death from HF	104 (4.5)	129 (2.8)	0.61 (0.47-0.79)	<0.001

These patients also had a lower rate of complex CV outcomes including myocardial infarction or death from other CV causes and hospitalization due to heart failure [10,12]. Before empagliflozin, other antihyperglycemic agents did not show a favorable CV profile [1,10] with a consistent benefit in patients with and without baseline heart failure [12]. Evidence suggests that the CV effects related to empagliflozin are a class effect, which would include dapagliflozin [11]. After the EMPA-REG OUTCOME results were published, some diabetes clinical practice guidelines [13] now suggest the use of SGLT2is, which have proven CV benefit, is a priority treatment in those T2DM patients who have not achieved glycemic targets and who have atherosclerotic CVD [7]. SGLT2is do not reduce stroke events [11].

SGLT2is have a higher incidence of genital infections and urinary infections as compared to other OAMs [6,8,10,11]. Vasilakou, et al. reported an imbalance in the incidence of breast and bladder cancer with dapagliflozin [6], thus requiring more studies of appropriate size and duration. The breast cancer (incidence rate ratio, 2.47) and bladder cancer (incidence rate ratio, 5.17) with dapagliflozin treatment may be due to an early diagnosis of preexisting cancer, a detection bias due to frequent urinalysis or chronic urinary tract infection [14]. When the cost efficacy of SGLT2is was measured, monotherapy with SGLT2is does not appear to be cost-effective compared with gliclazide or pioglitazone but may be competitive against sitagliptin [3]. More clinical trials on SGLT2i CV and renal safety are underway [7].

## Review

Dapagliflozin is a highly selective SGLT2i that controls blood glucose levels by blocking the SGLT2 receptors expressed in the S1 segment of the proximal kidney tubules leading to reduced renal glucose absorption [15]. Dapagliflozin has been shown to lower HbA1c, body weight, BP, and serum uric acid in clinical trials [16-18]. SGLT2is such as dapagliflozin affect visceral fat and blood glucose levels, both being components of metabolic syndrome and related to the progression of CVD. The mechanism of action by which dapagliflozin exerts its cardioprotective and nephroprotective effects is less understood and requires further research. Likewise, SGLT2i’s effect in a patient without T2DM but with decompensated heart failure may need to be explored [19]. Dapagliflozin is also indicated as monotherapy in patients who are intolerant to metformin or when metformin is contraindicated. As monotherapy, dapagliflozin is equivalent with metformin in reducing HbA1c levels [20,21]. Moreover, it can be used to complement other antihyperglycemic medications in patients with poor glycemic control [21]. Additional evidence is required to advocate dapagliflozin as monotherapy.

Dapagliflozin is mainly eliminated as its metabolite dapagliflozin 3-O-glucuronide (61%) in urine while <2% of the dapagliflozin dose is recovered in urine [15]. It has a half-life of 12.9 hours. In cases of renal and hepatic impairment, there is a higher systemic exposure to the drug. No clinically relevant differences were observed in dapagliflozin exposure with regards to age, race, sex, body weight, food, or presence of T2DM. In patients treated with dapagliflozin, urinary glucose excretion is dose-related [15], and dependent on plasma glucose levels as well as renal functions. A decrease in urinary glucose excretion was noticed due to the reduced filtered load in healthy individuals as compared to patients with T2DM [15].

When Dapagliflozin was co-administered with other commonly prescribed medications (e.g., simvastatin, valsartan, digoxin or warfarin) in patients with T2DM and CVD to assess drug interactions, dapagliflozin showed no drug interaction requiring dose adjustment; all the medications (including dapagliflozin) were well tolerated [15,22]. In another study on renal status, dapagliflozin was seen to improve hyperglycemia and facilitate weight loss in patients with T2DM by inducing controlled glucosuria with urinary loss of approximately 200 to 300 kcal/day [20]. Dapagliflozin treatment demonstrated no persistent, clinically significant osmolarity, volume, or renal status changes. There was no change in renal function. Serum uric acid decreased, serum magnesium increased, and serum phosphate increased at higher doses. Dose-related 24-hour urine volume and hematocrit increased minimally [20]. More recently, newly diagnosed patients with T2DM treated with SGLT2is had reduced atrial natriuretic peptide levels, which may benefit the CV system [23].

Common drug side effects include urinary tract infections, cystitis, and genital infections [17,20,24,25]. Hypoglycemic episodes were reported in 6% to 10% of patients [20,26]. Renal impairment and volume depletion with dapagliflozin are similar to that of placebo [24]. Dapagliflozin showed more adverse effects of hypotension, dehydration, genital infections, and renal impairment (in pre-existing mild/moderate renal disease) as compared to other OAMs. More research is needed to assess the efficacy and safety of dapagliflozin [26] regarding the renal profile.

Dapagliflozin reduces HbA1c similar to other OAMs, but it also has the beneficial effects on T2DM comorbidities [4,18]. CVDs are the most serious complication of T2DM. Hyperglycemia damages vessel endothelium which can lead to atherosclerosis. The resultant CVD is also the leading cause of morbidity and mortality in patients with T2DM [27]. Dapagliflozin is not associated with increased CV risk [28,29]. Since diabetic patients have an increased risk of CV event, the United States Food and Drug Administration now requires new diabetes therapies to show CV safety [30]. Moreover, few trials evaluate antihyperglycemic medications for older patients with T2DM or those with high-risk CV profiles. It is important to have additional evidence on effective therapies for high risk-patients and design therapies that provide not only good glycemic control but other clinical benefits as well [26].

Evidence suggests that patients with T2DM who were exposed to dapagliflozin had a lower risk of death from any cause irrespective of baseline CVD status. Therefore, dapagliflozin may be of benefit in those patients with a low risk of CVD [29]. Clinical trials have suggested that in patients with T2DM and high CV risk profiles, SGLT2i use was associated with reduced CVD and CV mortality compared with the use of other glucose-lowering drugs [31]. As compared with other glucose-lowering drugs, use of SGLT2i is linked with a decreased risk of severe hypoglycemia [31]. Dapagliflozin reduces systolic BP (−2.6 to −6.4 mmHg) with no clear dose relationship. There is a dose-related increase in hematocrit (1.5 to 2.9%) returning to baseline during follow-up. A mild increase in the blood urea nitrogen to creatinine ratio is also seen which returns to baseline during a four-week follow-up phase [20].

Dapagliflozin is well-tolerated and safe as noted in different cohorts including older patients, patients with poorly controlled diabetes, those with heart failure (New York Heart Association class II or higher) or CV risk factors [17,32]. In older patients with T2DM with preexisting CVD, dapagliflozin improves glycemic control and promotes weight loss. It is well-tolerated in these patients and does not increase hypoglycemic risk [17]. Dapagliflozin also improves glycemic control in patients with poorly controlled T2DM. It reduces body weight as well as systolic blood pressure in patients with poorly controlled hypertension and pre-existing CVD without affecting CV safety. It thereby improves risk factors for CVD [18,26]. These changes are also seen in T2DM patients with heart failure [32]. A meta-analysis involving more than 6000 patients also suggested that dapagliflozin is not associated with increased CV risk. This risk analysis involved >20% older patient population and patients with established CVD (>30%; Langkilde AM, Johansson P, Ptasynska A, Johnsson E: Abstract 11105: Cardiovascular safety of the SGLT2 inhibitor dapagliflozin: meta-analysis with >6000 patient-years exposure. Circulation. 2013, 128:A11105. http://circ.ahajournals.org/content/128/Suppl_22/A11105.short. Accessed September 5, 2017). Dapagliflozin has shown beneficial effects in older patients with CVD and hypertension and patients with a CVD history irrespective of varying degrees of CV risk. These favorable effects are the result of SGLT2 inhibition on CV risk factors. Furthermore, there was no increased risk of major adverse CV events (MACE; i.e., CV death, myocardial infarction, and stroke) in those patients who experienced a hypoglycemic event as compared with those who did not [28]. Dapagliflozin had favorable or neutral results in all individual types of CV events as compared to control-treated patients. Likewise, it appears to be beneficial in patients hospitalized for heart failure as compared to controls [28].

Compared to dipeptidyl peptidase-4 inhibitors, dapagliflozin had a lower risk of MACE, hospital events for heart failure, and all-cause mortality. Atrial fibrillation and severe hypoglycemia showed neutral associations [33]. Dapagliflozin is tolerable for long-term use as well. Its use over a period of two years maintains its effects on systolic BP reduction, glycemic control, and weight loss [24]. Likewise, treatment for longer duration has shown similar reduction in the incidence of myocardial infarction, stroke, CV death, and all-cause death, and also reductions in the incidence of end-stage renal disease, foot amputation, and diabetic retinopathy, when compared with the standard of care (e.g., diet and antihyperglycemic therapies) [16]. Hence adding dapagliflozin to currently available treatment options may decrease the CV and microvascular complications associated with T2DM [16].

Many more short-term and medium-term studies on dapagliflozin have proven CV safety [4] and benefits. One large ongoing study is DECLARE-TIMI58 (to be completed in 2019), which will give a more precise picture of the CV benefits. DECLARE-TIMI58 is an ongoing multicenter RCT to evaluate the effects of dapagliflozin on CV outcomes (e.g., the incidence of CV death, myocardial infarction or ischemic stroke) in T2DM patients with established CVD or CV risk factors (ClinicalTrials.gov identifier: NCT01730534) [4,21,35]. The DEFENCE trial evaluates the effect of dapagliflozin on the vascular endothelial function in patients with inadequately controlled T2DM. Dapagliflozin was used as add-on therapy to metformin for 16 weeks, and flow-mediated dilation on ultrasound was used to assess endothelial dysfunction. An improvement in flow-mediated dilation was seen in these patients. Until now, there is no concrete evidence suggesting the protective role of SGLT-2is on endothelial function or if they can suppress the progression of atherosclerosis [34]. Dapagliflozin may reduce oxidative stress which leads to its CV effects [34-36]. Another large RCT is the REFORM trial in Scotland, which has been ongoing since March 2015 to evaluate the safety and efficacy of dapagliflozin in patients with T2DM and known heart failure [2].

Dapagliflozin has shown reduced blood glycemic fluctuations without an increase in hypoglycemic episodes in patients newly diagnosed with T2DM on continuous glucose monitoring devices. It reduces oxidative stress as evident by a significant decrease in Plasma 8-PGF2α level (a biomarker of oxidative stress). Both the reduced glucose fluctuation and oxidative stress may benefit the CV system [36]. The lipid profile in patients treated with dapagliflozin showed minimal changes. There is a minor increase in both low-density and high-density lipoprotein cholesterol levels. However, more data are required to understand the significance of dapagliflozin on lipid levels [25,27].

The mechanism of action of dapagliflozin on CV events is not well understood. In a rodent model, it appears that changes in the mechanisms of Ca++ transport may be responsible for the negative inotropic effects of dapagliflozin in the ventricular myocytes [37]. Moreover, dapagliflozin in the rodent model has shown a delayed formation of diabetic atherosclerosis induced by a high-fat diet [27]. Dapagliflozin leads to a partial reversal of the formation of aortic atherosclerotic lesions in mice with diabetes mellitus. This effect is probably due to its inhibitory action on the secretion of interleukin-1β by macrophages via the reactive oxygen species-NLRP3-caspase-1 pathway [27]. In another rodent model, dapagliflozin significantly decreased macrophage infiltration. It reduced the gene expression of inflammatory cytokines and oxidative stress induced by hyperglycemia [38]. Evidence also suggests the nephroprotective action of dapagliflozin is due to its strong antihyperglycemic effect and suppression of inflammation, oxidative stress, and apoptosis in mice. It also reduces albuminuria. Hence, it may have potential in the management of diabetic nephropathy [38]. More research is required to explore and understand these effects in clinical settings. In Type 1 Diabetes Mellitus as well, dapagliflozin as an add-on treatment to insulin has shown improvement in glycemic control and lipid profile [39,40]. However, more trials of appropriate number and duration are required.

## Conclusions

Dapagliflozin is a relatively new and potent agent in the SGLT2i class of OAMs. Given its effects on HbA1c, CV safety profile, and its benefits as both monotherapy and combination therapy, dapagliflozin has significant potential as an antihyperglycemic medication that may offer additional therapeutic effects beyond blood glucose control, effects that may address common comorbidities seen in patients with T2DM. The mechanism of action for these benefits is currently unknown. More research is required to evaluate these benefits in clinical settings.
